# Novel Multi-Modal Therapies and Their Prognostic Potential in Gastric Cancer

**DOI:** 10.3390/cancers15123113

**Published:** 2023-06-08

**Authors:** Swathikan Chidambaram, Delia Cortés Guiral, Sheraz Rehan Markar

**Affiliations:** 1 Department of Surgery and Cancer, Imperial College London, London W2 1NY, UK; sc3312@ic.ac.uk; 2 Surgical Oncology and General Surgery Department, King Khaled Hospital, Najran 66262, Saudi Arabia; 3 Department of Molecular Medicine and Surgery, Karolinska Institutet, 17176 Stockholm, Sweden; 4 Nuffield Department of Surgery, University of Oxford, Oxford OX3 9DU, UK

**Keywords:** surgical oncology, gastric cancer, peritoneal carcinomatosis, hyperthermic intraperitoneal chemotherapy, HIPEC, pressurised intraperitoneal aerosolised chemotherapy, PIPAC

## Abstract

**Simple Summary:**

Gastric cancer with metastasis to the peritoneum carries a poor prognosis, with a 40% mortality rate. The optimal treatment modalities have not been established for gastric cancer patients with peritoneal carcinomatosis (GC/PC). We have conducted a systematic review and meta-analysis of available studies on novel treatment strategies including HIPEC and PIPAC to evaluate their safety and efficacy. Through this, our study contextualizes their role in the current treatment pathways for advanced gastric cancer.

**Abstract:**

Background: Gastric cancer has a poor prognosis and involves metastasis to the peritoneum in over 40% of patients. The optimal treatment modalities have not been established for gastric cancer patients with peritoneal carcinomatosis (GC/PC). Although studies have reported favourable prognostic factors, these have yet to be incorporated into treatment guidelines. Hence, our review aims to appraise the latest diagnostic and treatment developments in managing GC/PC. Methods: A systematic review of the literature was performed using MEDLINE, EMBASE, the Cochrane Review, and Scopus databases. Articles were evaluated for the use of hyperthermic intraperitoneal chemotherapy (HIPEC) and pressurised intraperitoneal aerosolised chemotherapy (PIPAC) in GC/PC. A meta-analysis of studies reporting on overall survival (OS) in HIPEC and comparing the extent of cytoreduction as a prognostic factor was also carried out. Results: The database search yielded a total of 2297 studies. Seventeen studies were included in the qualitative and quantitative analyses. Eight studies reported the short-term OS at 1 year as the primary outcome measure, and our analysis showed a significantly higher OS for the HIPEC/CRS cohort compared to the CRS cohort (pooled OR = 0.53; *p* = 0.0005). This effect persisted longer term at five years as well (pooled OR = 0.52; *p* < 0.0001). HIPEC and CRS also showed a longer median OS compared to CRS (pooled SMD = 0.61; *p* < 0.00001). Three studies reporting on PIPAC demonstrated a pooled OS of 10.3 (2.2) months. Prognostic factors for longer OS include a more complete cytoreduction (pooled OR = 5.35; *p* < 0.00001), which correlated with a peritoneal carcinomatosis index below 7. Conclusions: Novel treatment strategies, such as HIPEC and PIPAC, are promising in the management of GC/PC. Further work is necessary to define their role within the treatment algorithm and identify relevant prognostic factors that will assist patient selection.

## 1. Introduction

Gastric cancer (GC) is the fifth most common cause of malignancy and the fourth leading cause of cancer-related mortality globally, with an estimated incidence of 1.1 million cases that contribute to nearly 800,000 deaths annually [1]. Large-scale epidemiological studies have estimated synchronous peritoneal carcinomatosis (PC) in up to 40% of patients. This significantly reduces the median survival to 3−6 months and the 5-year survival rate to 0%. Compared to other gastrointestinal malignancies, GC has the highest load of peritoneal disease, likely due to spread via direct contact with tumour cells [2]. In fact, in patients who underwent initial curative resection, recurrence in the peritoneum accounts for over 40% of cases [3]. Historically, GC/PC has not been considered for surgical resection [4]. Following the REGATTA trial, the Japanese Gastric Cancer Association does not recommend surgical approaches to manage GC/PC. Similarly, the National Comprehensive Cancer Network (NCCN) guidelines recommend systemic chemotherapy or best supportive care for GC with peritoneal dissemination [5,6,7]. In contrast, the ESMO guidelines support cytoreductive surgery in selected patients with peritoneal spread [8]. Hence, there is a lack of consensus on the optimal management of this cohort of patients.

Currently, the management of GC/PC is undergoing notable changes with new discoveries in both diagnostic and treatment modalities. Typically, patients with GC/PC are diagnosed and confirmed based on peritoneal washings, which give a measurement of the disease burden through the peritoneal carcinoma index (PCI) [9]. However, there are increasing opportunities to combine this with novel diagnostics, including molecular assays. Furthermore, there are novel ways of delivering conventional chemotherapy agents that have proven successful in other GI cancers and can also be applied to GC/PC. These include hyperthermic intraperitoneal chemotherapy (HIPEC), which delivers heated chemotherapy agents directly into the peritoneal cavity, and pressurised intraperitoneal aerosolised chemotherapy (PIPAC), where chemotherapy is administered at high pressures within the abdomen. Within the literature, there is a paucity of work summarising the evidence for these novel treatment strategies as well as prognostic factors that aid patient selection for those who will benefit from these therapies. Hence, our review aims to provide an update on the latest diagnostic and treatment developments in managing GC/PC, their limits, and how these can be overcome.

## 2. Methods

Literature search methods, inclusion and exclusion criteria, outcome measures, and statistical analysis were defined according to the Preferred Reporting Items for Systematic Reviews and Meta-Analyses (PRISMA) guidelines. Patients were not involved in the conception, design, analysis, drafting, interpretation, or revision of this research. Hence, ethical approval was not required and thus not sought for this study.

### 2.1. Search Strategy

The following databases were searched: MEDLINE (1946 until the first week of February 2022) via OvidSP; MEDLINE in-process and other non-indexed citations (latest issue) via OvidSP; Ovid EMBASE (1974 to the latest issue); and Scopus (1996 until the present). The last search was performed in February 2022. In order to capture all studies evaluating the use of multimodal strategies in peritoneal carcinomatosis in gastric cancer, we used three different search strategies:

1. ‘laparoscopy’, ‘peritoneal cytology’, ‘cancer staging’, and ‘prognosis’, “gastric” and “stomach”, combined with “cancer or tumo?r* or adenocarcinoma or neoplasm”.
2. “gastric” and “stomach” combined with “cancer or tumo?r* or adenocarcinoma or neoplasm”, “peritoneal carcinomatosis”, carcinomato* or carcino* or metast* or neoplas*”, “HIPEC”, “IPHP”, “IHCP”, “CHPP”, “hyperthermic intraperitoneal chemotherapy”, “intraperitoneal hyperthermic perfusion”, “intraperitoneal hyperthermic chemoperfusion”, “continuous hyperthermic peritoneal perfusion”, “cytoreductive surgery”, “cytoreduction”, “CRS”, “prognosis”, “survival”, “survival rate”, and “risk ratio”.
3. “gastric” and “stomach” combined with “cancer or tumo?r* or adenocarcinoma or neoplasm”, “peritoneal carcinomatosis”, “carcinomato* or carcino* or metast* or neoplas*”, (pressur* or laparoscopic*); (intra-periton* or intra?periton* or “intra periton*” or intra-abdominal* or intra?abdominal or “intra abdominal*”); (chemo?therap* or chemo or therap* or treat*); PIPAC* or ePIPAC* or PITAC*. The three strings were then combined using the AND modifier.

The first search was aimed at identifying the evidence for the prognostic value of peritoneal cytology and is an update to our earlier work evaluating the use of chemotherapy to downstage GC with PC to cytology-negative cancer and determine if this influenced survival. The second and third searches were aimed at evaluating the efficacy of HIPEC and PIPEC in improving the prognosis of GC with PC. References of included articles were screened, and a hand search was performed to identify missing articles. Two reviewers (SC and SRM) independently assessed the titles and abstracts for the inclusion of relevant references. In cases where there was disagreement over inclusion, a third author (DCG) was consulted.

### 2.2. Selection of Studies

Studies were included if they had investigated the use of chemotherapy regimens in the management of gastric cancer with peritoneal metastasis. Chemotherapy strategies include standard regimens, i.e., HIPEC and PIPEC. Studies also had to report survival outcomes and, where possible, measures of morbidity and recurrence. The study design was restricted to only randomised clinical trials (RCTs) and prospective cohort studies. Studies were restricted to a publication date of 2022 to keep abreast of the latest treatment strategies. Studies were excluded if they did not investigate the aforementioned oncological therapies in GC with PC, did not report the survival outcomes, had incomplete data on outcome measures, or were not in the English language. Studies with incompatible designs, including case series, letters, comments, and reviews, were also excluded.

### 2.3. Outcome Measures and Data Extraction

Our main aim was to assess the impact of various modes of chemotherapy on the survival outcomes of patients with GC and PC. Hence, the main outcome measures included overall survival, recurrence-free survival, and disease-free survival. We were also interested in the cytology results after undergoing chemotherapy (HIPEC or PIPAC). In addition, we collected study meta-data (first author, year of publication, study design, sample size); relevant patient demographic data (age, sex, and comorbidities); pathological characteristics (stage, grade, size, PCI); oncological treatment details (duration, agents used, and number of cycles); and surgical intervention (operation technique or approach).

### 2.4. Quality Assessment of Selected Studies

Two reviewers (SC and SRM) assessed the quality of each included study by independently evaluating the risk of bias using the Cochrane Risk of Bias tool (RoB) for randomised controlled trials. The RoB tool was designed by epidemiologists and statisticians to appraise RCTs on six domains of bias: selection bias, performance bias, detection bias, attrition bias, reporting bias, and other bias. The results of an assessment of the risk of bias are typically presented in a tabular format with justifications as required.

### 2.5. Statistical Methods

Review Manager 5.3 (Cochrane Collaboration, Oxford, UK) was used for statistical analysis of the data. Two types of modelling were used to assess the heterogeneity of the data: fixed-effects and random-effects. Data is given as odds ratios and 95% confidence intervals (CI) for all non-continuous data and as standardised mean differences and 95% CI for all continuous data. Where studies had reported medians and ranges, the data was transformed to means and standard deviations using the methods published by Hozo et al. [10]. In all cases, statistical heterogeneity was assessed by using the I^2^ statistic and was categorised as low, moderate, and high for an I^2^ statistic above 25%, 50%, and 75%, respectively. Results above 60% were considered substantial heterogeneity.

## 3. Results

### 3.1. Search Results

**Figure 1:**
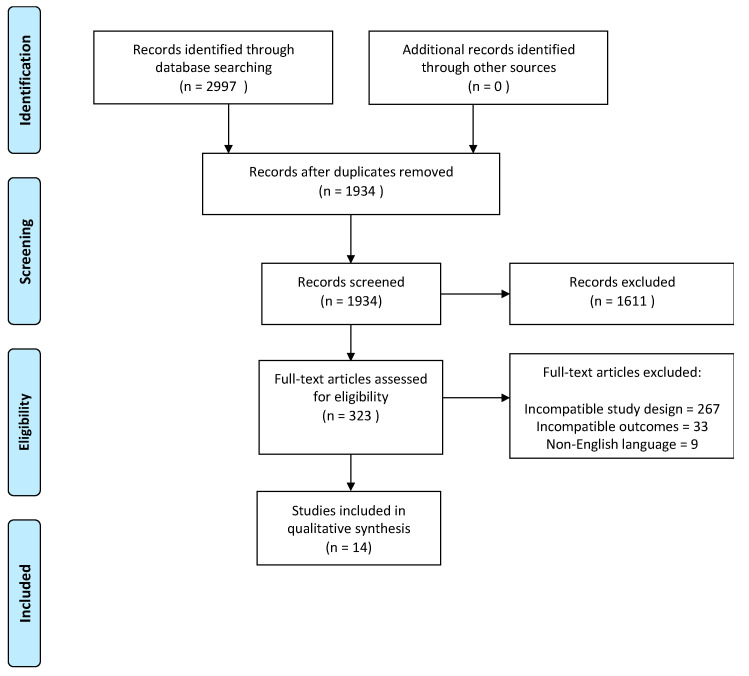
PRISMA 2020 flow diagram.

The database search yielded a total of 2297 studies. After duplicates were removed, the titles and abstracts of the remaining 1934 studies were assessed for eligibility, and 1625 studies were removed. A further 296 studies were excluded after full-text review due to incompatible outcome measures or study designs. Fourteen studies that reported on overall survival (OS) outcomes were included in the qualitative and quantitative analyses. Most of the excluded studies were deemed not eligible due to incompatible study design (retrospective cohort studies, case series, case reports, abstracts, letters, and comments); inclusion of heterogeneous cohorts (gastric cancer with distant metastasis and treatment for palliative intent); missing data on survival outcomes; and overlapping treatment strategies. The flow of study selection is shown in Figure 1 [11].

### 3.2. Patient and Study Characteristics

Seventeen studies were included in this study. Ten studies were randomised controlled trials that compared HIPEC and CRS with only CRS [12,13,14,15,16,17,18,19,20,21] (Table 1). Three of the studies prospectively evaluated the use of PIPAC in gastric cancer patients [22,23,24] (Table 2). All studies recruited patients with gastric cancer for treatment with curative or prophylactic intent and reported on overall survival (OS), with three studies reporting median OS and nine studies reporting mortality rates at one and five years. The total sample size of patients included in the quantitative analysis was 1058. The range of ages of the patients included was 24–74, while the total number of males was 525. Surgical intervention was carried out using an open approach in all studies. The most common agents used for HIPEC were MMC and oxaliplatin. Similarly, oxaliplatin was most commonly used in PIPAC sessions. The full characteristics of the studies are shown in Table 1 and Table 2. Furthermore, the quality of studies was generally good based on the Cochrane RoB tool with minimal bias (Table 3).

### 3.3. HIPEC and Overall Median Survival

**Figure 2:**
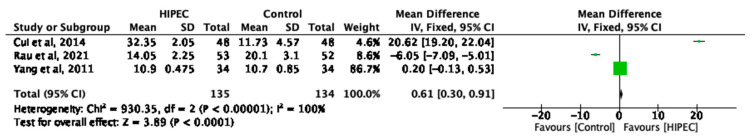
HIPEC and overall median survival [16,18,20].

Three of the included studies reported the median length of OS. The total sample size was 269, with similar numbers for HIPEC/CRS (*n* = 135) and CRS (*n* = 134). There was evidence of significant heterogeneity between studies (I^2^ = 100%, *p* < 0.00001), although this is likely due to the small number of studies included in this analysis. The analysis demonstrated a longer OS survival with HIPEC and CRS compared to CRS (pooled SMD = 0.61; 95% CI 0.30, 0.91, *p* < 0.00001) (Figure 2).

### 3.4. HIPEC and Short-Term Survival

**Figure 3:**
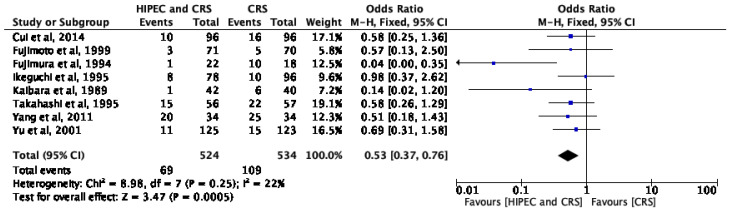
HIPEC and short-term survival [12,13,14,15,16,17,18,20].

Eight studies reported the short-term OS at 1 year as the primary outcome measure. The total sample size was 1058, with 524 patients in the HIPEC/CRS arm and 534 patients in the CRS arm. In total, there were 69 and 109 deaths in the arms, respectively. There was evidence of minimal heterogeneity between studies (I^2^ = 22%, *p* = 0.0005), and fixed-effects analysis showed a significantly higher OS for the HIPEC/CRS cohort compared to the CRS cohort (pooled OR = 0.53; 95% CI 0.37, 0.76, *p* = 0.0005) (Figure 3).

### 3.5. HIPEC and Long-Term Survival

**Figure 4:**
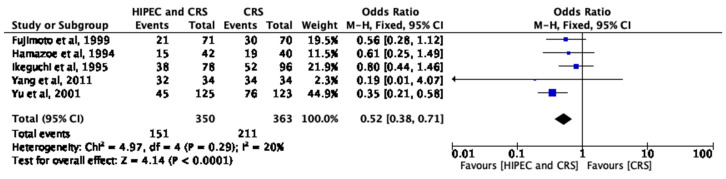
HIPEC and long-term survival [13,14,17,20,21].

Five studies reported the long-term OS at 5 years as the primary outcome measure. The total sample size was 713, with 350 patients in the HIPEC/CRS arm and 363 patients in the CRS arm. In total, there were 151 deaths in the HIPEC/CRS arm, compared to 211 deaths in the CRS arm. There was evidence of minimal heterogeneity between studies (I^2^ = 20%, *p* < 0.0001), and fixed-effects analysis showed a significantly higher OS for the HIPEC/CRS cohort compared to the CRS cohort (pooled OR = 0.52; 95% CI 0.38, 0.71, *p* < 0.0001) (Figure 4).

### 3.6. PIPAC and Median Survival

Three of the included studies on PIPAC reported the median length of OS. The total sample size was 147. All three studies were prospective, single-arm trials. Altogether, pooled analysis showed a mean overall survival of 10.3 months (pooled SD = 2.16).

### 3.7. Completeness of Cytoreduction, PCI, and One-Year Survival

**Figure 5:**
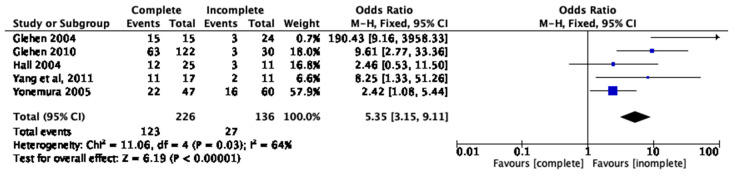
Cytoreduction and short-term survival [20,25,26,27,28].

Five studies compared the short-term OS at 1 year between patients who achieved complete cytoreduction and those who did not achieve this [20,25,26,27,28]. The total sample size was 363, with 226 patients in the complete cytoreduction group and 136 in the incomplete group. In total, there were 123 and 27 deaths in the arms, respectively. There was evidence of moderate heterogeneity between studies (I^2^ = 64%, *p* = 0.03), and fixed-effects analysis showed a significantly higher OS for the complete cytoreduction cohort compared to the incomplete cytoreduction cohort (pooled OR = 5.35; 95% CI 3.15, 9.11, *p* < 0.00001) (Figure 5).

Given the variety of formats in which PCI was reported, it was not possible to generate a pooled PCI, but studies reported a higher OS with higher cytoreduction and lower PCI. Yonemura et al. demonstrated CC-0 and CC-1 in 91% of patients with a PCI of 6 and below, but only 42% with a PCI of above 7. The CYTO-CHIP study showed that long-term survival was minimal if PCI was above 13, while the longest median survival of 22.8 months was noted for PCI of 0, which correlated with CC-0. Glehen et al. stratified their patients into four groups: 1–6, 7–12, 13–19, and >19. The mean PCI was 13.1 (8.9), and survival was higher for the PCI 1–6 cohort at 49 months compared to 10 months if PCI was above 19.

## 4. Discussion

Our analysis of recent RCTs comparing CRS and CRS/HIPEC for gastric cancer with and without PC confirmed an overall survival benefit in the short and long term. This is concordant with a previous meta-analysis by Desiderio et al., although that study was limited due to significant heterogeneity in patient cohorts, tumour-level characteristics, and treatment regimens [29]. Furthermore, in the most recent GASTRIPEC trial, Rau et al. reported better progression-free survival with CRS and HIPEC compared to just CRS [18]. Given the morbidity of systemic therapies, patient selection is key to ensuring maximal survival benefit, and factors associated with a lower disease burden, such as lower PCI, more complete cytoreduction, optimal preoperative performance status, synchronous PC, and response to neoadjuvant chemotherapy, should favour the use of HIPEC [30,31]. In this regard, both diagnostic laparoscopies and peritoneal cytology play a crucial role in identifying patients with favourable factors.

PIPAC is another modality that has gained attention since initial work showed a 50% tumour response in patients with GC/PC. By using high pressure to deliver heated and aerosolised chemotherapy, PIPAC allows for enhanced coverage and drug uptake [32]. Although the included studies did not have a comparative arm, they did report better survival and quality of life despite recruiting patients with a heavy disease burden. Our pooled analysis confirmed these results and is consistent with prior work. For example, Nadiradze et al. reported a median survival of 15.4 months even in patients with high-risk features, such as signet ring histology and a high PCI of 16 [33]. In another study, Di Giorgio et al. found a longer median survival in patients undergoing more than one session of PIPAC [34]. The correlation between the variables in PIPAC, such as the number of sessions and agents used, requires further work. Given the heterogeneity in patient cohorts, it remains unknown if bidirectional therapy with PIPAC combined with NAC has any added benefit. Nevertheless, PIPAC has been established as a safe modality following the standardisation of protocols and adherence to them [32]. In one study, Alyami et al. reported the downstaging of initially unresectable GC/PC with PIPAC to subsequently facilitate CRS and HIPEC [35]. Compared to CRS/HIPEC, PIPAC also carries a lower morbidity (CTCAE grade III/IV complication rate of 52.3%) [36]. Given these findings, PIPAC requires further evaluation as a first-line alternative at an earlier point in a patient’s treatment journey, when they may have a better performance status.

In studies evaluating HIPEC and PIPAC, the peritoneal cancer index (PCI) serves as a useful quantitative assessment of cancer distribution that combines both peritoneal implant size and the distribution of nodules on the peritoneal surface [37]. PCI is inversely related to complete cytoreduction (CCR), with some studies reporting a 79% decrease in CCR as the PCI increased from 6 to above 13 [38]. This has a significant implication for survival, given that our analysis showed that survival was five times higher with complete cytoreduction compared to incomplete reduction. This is consistent with a previous meta-analysis by Coccolini et al., who reported that patients with CC-0 and CC-1 had better outcomes at 5-year follow-ups compared to CC-2 or CC-3 cohorts [39]. Nevertheless, the exact values for a prognostic PCI remain to be established. In this paper, PCI was reported in various formats, which limited any pooled analysis that could be performed. For example, Yang et al. used a cut-off score of 20 to distinguish between low and high PCI, while others have used a cut-off of 12 [20,25]. Yonemura et al. demonstrated CC-0 and CC-1 in 91% of patients with a PCI of 6, but only 42% with a PCI of 7, which has been supported by other studies as well [38,40]. PCI of 7 is strengthened by the recent CYTO-CHIP study, which showed PCI to be the second most important factor in a cohort of 277 patients, the largest cohort of patients treated with complete CRS with/without HIPEC [41]. Not only was long-term survival rare in patients with a PCI above 13, but the mean PCI in patients with better survival was 7.2. In the ongoing RENAISSANCE and SURGIGAST trials, the inclusion criteria for PCI are also set at 7 and will finally provide robust trial evidence for this value as an important prognostic factor [42].

In the absence of consensus regarding the optimal treatment strategy for M1 disease, a better understanding of prognostic factors to aid patient selection is crucial. Of these, the status of peritoneal cytology and macroscopic disease are the most prominent considerations. In a previous meta-analysis, our group highlighted that patients with negative peritoneal cytology had a higher survival rate compared to those with positive cytology prior to treatment, and macroscopic peritoneal dissemination was a poor prognostic factor, as shown by our meta-analysis [43]. However, if the cytology status were modified by chemotherapy from a positive to a negative, this would result in better overall survival. Conversely, a change in cytology status from negative to positive results in a worse prognosis by as much as 25% [44]. In their study, Aizawa et al. showed that in patients who converted from positive to negative after the induction chemotherapy and underwent surgery, the median survival time of 30.4 months and 5-year survival rate of 34.6% were higher than the corresponding values of 15.0 months and 17.6% [45]. In their multivariate analyses of gastric cancer patients with positive cytology but no gross peritoneal metastasis, Shim et al. report the absence of chemotherapy as the strongest clinical factor for poorer disease-free survival [46]. Nakamura et al. and Yasufuku et al. confirmed that similar survival outcomes were achieved in patients who underwent surgery if they had clearance of macroscopic disease after chemotherapy confirmed at second-stage laparoscopies [47,48]. Taken together, both negative peritoneal cytology status and clearance of macroscopic disease are important prognostic factors in M1 gastric cancer patients that are modifiable by neoadjuvant therapies.

Although cytology is an important modifiable prognostic factor, the analysis and processing of peritoneal washings are not standardised and vary by centre. The sensitivity, specificity, and overall accuracy of conventional methods based on Papanicolau or Giemsa staining in identifying recurrence have been estimated at 11–80%, 86–100%, and 73–92%, respectively [49]. Given the low sensitivity of conventional methods, patients with negative cytology might actually experience rapid recurrence after curative surgery. In these situations, the use of molecular markers, such as carcinoembryonic antigen (CEA), cytokeratin 19 (CK-19), and cytokeratin 20 (CK-20), is better correlated with peritoneal recurrence and associated with adverse outcomes. Most recently, one-step nucleic acid amplification (OSNA) has been applied to detect CK-19 mRNA in peritoneal washings with a sensitivity and specificity of 83.3% and 87.8%, respectively. In their study, Geca et al. reported a 30.5% detection rate using OSNA, which was significantly higher than 7.3% using conventional methods [50,51]. Even in cytology-negative patients, positive molecular status almost doubles the risk of a poor prognosis, so OSNA provides additional modifiable prognostic information that otherwise would not be possible with standard methods [52]. In another study, Kumagai et al. analysed 394 lymph nodes from 61 patients and showed a concordance rate, sensitivity, and specificity of 94.2%, 83.3%, and 95.9%, respectively. Intraoperative OSNA assays were as accurate in detecting lymph node metastasis as histological examination of blocks of 2 mm sections [53]. Although OSNA has better sensitivity, conventional methods are simpler to perform and remain the standard in most centres for practical reasons.

## 5. Future Work

Further results from ongoing international, multi-centre RCTs are necessary to define the efficacy and safety of HIPEC for both prophylactic and curative purposes. The GASTRICHIP trial has enrolled 306 patients with disease involving the serosa with or without lymph node involvement and positive cytology at peritoneal washing [54]. The PERISCOPE II trial extends this further and is evaluating HIPEC in patients with primary T3-T4 gastric tumours, including lymph nodes, limited peritoneal dissemination, or positive peritoneal cytology [55]. Lastly, the DRAGON II trial is investigating the use of neoadjuvant laparoscopic HIPEC, D2 curative gastrectomy, and intraoperative prophylactic HIPEC in T4 gastric cancer with serosal involvement and absence of peritoneal carcinomatosis. These studies will define prognostic factors and determine the role of HIPEC in gastric cancer. Currently, the GASPACCO trial is a single-centre, randomised, phase 3 trial evaluating the efficacy of PIPAC in preventing PC in patients with locally advanced GC. Other trials are evaluating the combined use of PIPAC with other therapies in treating GC/PC, including the use of immunotherapy agents, such as nivolumab [56,57].

## 6. Conclusions

Overall, the management of patients with GC/PC is an evolving field with growing evidence for novel treatment strategies, such as HIPEC and PIPAC. While our work highlights the survival benefits of HIPEC and PIPAC, large scale RCTs are ongoing to fully define their role in the treatment algorithm, specifically when combined with existing treatment modalities. It is clear that any survival advantage gained will ultimately be due to careful patient selection, so a better understanding of prognostic factors from future work is crucial to characterising the ideal patient cohort.

## Figures and Tables

**Table 1:** Study and patient characteristics of studies evaluating HIPEC and CRS.

Study	HIPEC/CRS (n)	CRS (n)	Age (HIPEC; CRS)	Sex	T2 (%)	T3–T4 (%)	N0–N1		HIPEC Characteristics		
									Agents	Temperature (°C)	Duration (mins)
Kaibara et al. (1989)	42	40	-	-	-	100	-	-	MMC	44–45	60
Fujimura et al. (1994)	22	18	60.2 63.2	12 9	45	55	23	16	MMC CDDP	41	60
Hamazoe et al. (1994)	42	40	56.5 (10.4) 63.4 (9.6)	25 31	19.5	80.5		26	MMC	48	60
Ikeguchi et al. (1995)	78	96				100			MMC	44–45	60
Takahashi et al. (1995)	56	57	54.5 55.7	34 34	-	-	-	-	MMC		180
Fujimoto et al. (1991)	71	70	58.5 (8.1) 59.2 (9.1)	50 51	17	83	11	130	MMC	45	120
Yu et al. (2001)	125	123	54 55	84 81	30.6	69.4	165	83	MMC 5-FU	37	
Yang et al. (2011)	34	34	50 (24–74) 51 (28–75)	16 19	-	-	-	-	CDDP MMC	43	60–90
Cui et al. (2014)	96	96	39–72 39–70	22 21	-	-	-	-	CDDP 5-FU	41–43	90
Beeharry et al. (2019)	40	40	59 (10) 58 (10)	23 23		100	27	53	CDDP	42	60
Rau et al. (2015)	53	52	-	-	-	-	-	-	MMC CDDP	42	60

**Table 2:** Study and patient characteristics of studies evaluating PIPAC.

Study	Age	Sex	Number of PIPAC Sessions	Chemotherapy Used for PIPAC	Interval between PIPAC Sessions	Bidirectional with SACT (%)	Systemic Regimen	Median OS (Months)	One-Year OS (%)
Khomyakov et al. (2016)	-	-	56	Cisplatin Doxorubicin	6 weeks	Yes (100%)	XELOX	13.0	49.80%
Struller et al. (2019)	55.1 (13)	10	43	Cisplatin (7.5) + Doxorubicin (1.5)	6 weeks	No	N/A	NS	NS
Ellebᴂk et al. (2020)	58.5 (31–70)	7	52 (11 ePIPAC)	Cisplatin (7.5) + Doxorubicin (1.5)	4–6 weeks	Yes (*n* = 9, 45%)	NS	11.5	NS

**Table 3:** Risk of bias assessment for RCTs.

Study	Random Sequence Generation	Allocation Concealment	Blinding of Participants and Personnel	Blinding of Outcome Assessments	Incomplete Outcome Data	Selective Reporting	Other Biases
Kaibara et al. (1989)	+	+	+	+	-	+	?
Fujimura et al. (1994)	+	+	+	+	-	+	?
Hamazoe et al. (1994)	+	+	+	+	-	-	?
Ikeguchi et al. (1995)	+	+	+	+	-	-	?
Takahashi et al. (1995)	+	+	+	+	+	-	?
Fujimoto et al. (1991)	+	+	+	+	+	-	?
Yu et al. (2001)	+	+	+	+	+	+	+
Yang et al. (2011)	+	+	+	+	+	+	+
Cui et al. (2014)	+	+	+	+	+	+	+
Beeharry et al. (2019)	+	+	+	+	+	+	+
Rau et al. (2015)	+	+	+	+	+	+	+
Khomyakov et al. (2016)	+	+	+	+	+	+	?
Struller et al. (2019)	+	+	+	+	+	+	?
Ellebᴂk et al. (2020)	+	+	+	+	+	+	?

## Data Availability

Not applicable.
